# Dietary diversity and nutritional adequacy among married Filipino immigrant women: The Filipino Women’s Diet and Health Study (FiLWHEL)

**DOI:** 10.1186/s12889-018-5233-z

**Published:** 2018-03-15

**Authors:** Grace P. Abris, Na-Hui Kim, Sherlyn Mae P. Provido, Sangmo Hong, Sung Hoon Yu, Chang Beom Lee, Jung Eun Lee

**Affiliations:** 1 Department of Food and Nutrition, Sookmyung Women’s University, Cheongpa-ro 47-gil 100, Yongsan-gu, Seoul, 04310 Korea; 2 Division of Endocrinology, Department of Internal Medicine, Hallym University Dongtan Sacred Heart Hospital, 7, Keunjaebong-gil, Hwaseong-si, Gyeonggi-do, Seoul, 18450 Korea; 3 Division of Endocrinology and Metabolism, Department of Internal Medicine, Hanyang University Guri Hospital, Hanyang University College of Medicine, 153 Gyeongchun-ro, Guri, 11923 Korea; 4 Department of Food and Nutrition, College of Human Ecology, Research Institute of Human Ecology, Seoul National University, 1 Gwanak-ro, Gwanak-gu, Seoul, 08826 Korea

**Keywords:** Food variety, Migrants’ health, Adequate nutrition

## Abstract

**Background:**

Migration has an influence on health behavior and food intake. Dietary variety is a key component to high-quality diets because a single food item does not contain a variety of nutrients and may not reflect nutritional adequacy. We aimed to compare the dietary diversity scores (DDS), food variety scores (FVS), and nutrient adequacy levels of married Filipino immigrant women in Korea to those of Korean women.

**Methods:**

We matched the data of 474 participants aged 20-57 years from the Filipino Women’s Diet and Health Study (FiLWHEL) by age category with those of married Korean women randomly selected from the Korea National Health and Nutrition Examination Survey (KNHANES). Dietary information in FiLWHEL and KNHANES were assessed through the 24-hour recall method. We calculated the DDS by summing the number of eleven food groups consumed (DDS 10 g if they consumed at least 10 g/day; DDS all if they consumed any amount) and the FVS by counting the number of food items consumed. For nutrient adequacy, we calculated the probability of adequacy (PA) and intake below the estimated average requirement (EAR).

**Results:**

Filipino women had a lower DDS and FVS in comparison to Korean women. The means (±SDs) of DDS 10 g, DDS all, and FVS for Filipino women versus Korean women were 6.0 (±1.6) versus 6.8 (±1.5) (*p* < 0.001), 6.7 (±1.7) versus 7.9 (±1.4) (*p* < 0.001) and 9.2 (±3.3) versus 14.7 (±4.9) (*p* < 0.001), respectively. When we compared each food group, the intakes of fish, other seafood, legumes/seeds/nuts, eggs, vegetables, and fruits were lower for Filipino women than for Korean women. The mean probability of adequacy (MPA) of nutrient intake of the nine selected nutrients was lower for Filipino women in comparison to Korean women. The mean (±SD) was 0.55 (±0.28) versus 0.66 (±0.26), respectively.

**Conclusions:**

Our findings showed that married Filipino immigrant women in Korea had lower dietary variety scores in comparison to Korean women. This was reflected in their nutritional adequacy. Nutrition education focusing on the promotion of eating a variety of foods may be needed for Filipino immigrant women in Korea.

**Electronic supplementary material:**

The online version of this article (10.1186/s12889-018-5233-z) contains supplementary material, which is available to authorized users.

## Background

The number of married female immigrants in Korea has grown considerably since the 1990s [1]. As of 2014, they represented 8.0% of the total marriages and 4.9% of all live births could be accounted for in this population [2]. In 2014, Filipinos ranked fourth after the Chinese, Vietnamese, and Japanese. As of 2014, there were 10,736 married Filipino immigrant women in Korea [3]. Previous studies showed that Filipino immigrants have higher health risks, morbidities, and mortality rates in comparison to other immigrant groups and/or local hosts [4–10]. Married Filipino immigrant women in Korea (22%) have a higher prevalence of obesity (BMI ≥ 25 kg/m^2^) in comparison to the Chinese (16.7%), Vietnamese (7.8%), and other groups (19.2%) [4]. In the United States, Filipinos have the highest prevalence of obesity (14%) in comparison to Asian Indian (6%), Vietnamese (5%), and Chinese adults (4%) [5, 6]. In the US, Filipinos also have a higher breast cancer mortality and a higher prevalence of diabetes and hypertension in comparison to the Chinese, Vietnamese, Korean, Japanese, Caucasian, and African-American groups [7–10].

A study specifically on the married immigrant population in Korea is of interest because of the different experiences that likely affect their diet and health. Difficulties in language and adaptation to new foods and the environment, loneliness, and conflict with their husbands and mothers-in-law [11, 12] may be some of the stressful factors that married immigrants face. Furthermore, women at the reproductive age are often nutritionally at risk because of their physiological needs during pregnancy and lactation [13].

Cultural influences affect health behavior and health-related risks. Dietary habits are formed early in life and mostly continue until adulthood [14, 15]. However, migration changes dietary behavior. Following immigration in the US, Chinese Americans have increased their intake of Western foods and food diversity but have decreased their intake of traditional Chinese foods [16]. Chinese Americans who have lived in the US for many years have increased their consumption of vegetables, fats/sweets, and beverages [16]. Therefore, we propose that Filipino married immigrant women in Korea have adopted the food habits of their host country, as other studies have suggested [17–19]. However, some homeland dietary practices may have been maintained. In the Philippines, the diet is composed of rice, fish, and meat, with a small serving of either fruits or vegetables [20]. Korea’s traditional diet is more varied and mainly composed of rice, soup, kimchi, soybean products, raw or steamed vegetables, and other various side dishes [21–23]. Dietary diversity is a key component to high-quality diets. Dietary guidelines in many countries have emphasized a variety of foods [24–27] because a variety provides essential nutrients that cannot be found in a single food item. Several studies have shown that dietary variety is positively associated with nutrient adequacy [28, 29] and reduced all-cause mortality risk [30] as well as a decrease in chronic diseases, including cardiovascular disease (CVD) [31], type 2 diabetes [32], and several types of cancer [33–38]. Studies among Korean adults have also found that dietary variety is significantly associated with the quality of nutrient intake [39, 40]. In these studies, there was an inadequate nutrient intake of calcium and iron [39] and an inadequate food intake, especially of the dairy and fruit groups [40]. In the Philippines, nutritional guidelines for Filipinos have been revised based on the results of the 2008 National Nutrition Survey. One of the revisions was an emphasis on a more varied diet [41]. The report indicated that protein, iron, vitamin A, vitamin C, calcium, thiamin, riboflavin, and niacin were below the 100% estimated average requirement (EAR) in a representative sample of Filipino adults in the Philippines [20]. Given the evidence that Filipinos have a high risk of chronic diseases in other receiving countries and that nutritional inadequacy is prevalent in the Philippines, we aimed to compare the dietary diversity score (DDS), food variety score (FVS), and nutritional adequacy of married Filipino immigrant women in Korea to those of Korean women.

## Methods

This study is reported according to the Strengthening the Reporting of Observational Studies in Epidemiology-Nutritional Epidemiology (STROBE-nut) guidelines [42, 43].

### Study population

The Filipino Women’s Diet and Health Study (FiLWHEL) comprises a cohort of Filipino women married to Korean men in Korea [44]. FiLWHEL collects comprehensive health and dietary information based on a convenience sampling method. We visited cities in Korea including Seoul, Incheon, and Daejeon and several parts of Gyeonggi and Chungcheong Provinces. The specific sites for data collection were at universities, university hospitals, community centers, and churches. Filipino community leaders played an important role in the recruitment of participants. We disseminated advertisements through personal contacts and social media. Filipino women were invited to enroll in the study if they were ever married to Korean men and if they were 19 years old or older. We collected baseline data for this cohort from March 2014 to April 2016, which included demographic information, immigration-related questions, health-related behaviors, medical history, quality of life, children’s health information, anthropometric data, and biospecimens including toenails, blood, and DNA. In total, we enrolled 504 Filipino women in this study, and 497 Filipino women provided information from a 24-hour recall through an in-person interview administered by Filipino research staff. All interested participants provided signed informed consent to participate. This study was approved by the Institutional Review Board of Sookmyung Women’s University (reference number SMWU-1311-BR-012). Details of the design and methods of FiLWHEL are published elsewhere [44].

To compare the dietary diversity of Filipino women to that of Korean women, we randomly selected married Korean women from the Korea National Health and Nutrition Examination Survey (KNHANES) 2012-2014. Since 1998, KNHANES has been a national surveillance system for Koreans that assesses their health and nutritional status. KNHANES is a yearly nationwide cross-sectional survey, which includes approximately 10,000 nationally representative non-institutionalized civilians [45]. Both FiLWHEL and KNHANES have collected available dietary intake information using the one-day 24-hour recall through an in-person interview. For FiLWHEL, we and our participants estimated portion sizes using food miniatures, photographs, household measures, weight/volume, and standard units and portions. We computed nutrient values derived from the 24-hour recall data of FiLWHEL using the Computer Aided Nutritional Analysis version 4.0 released by the Korean Nutrition Society [46]. However, when food items could not be obtained from the software, we used nutrition information from the food composition tables of the Food and Nutrition Research Institute of the Philippines [47] (especially for Filipino food), Korean Rural Development Administration [48], US Department of Agriculture [49], or directly from the manufacturer. To ensure data quality, all interviews were administered by Filipinos who could communicate in the Filipino language, and all study periods were supervised. Furthermore, all records were double-checked before and after data entry [44]. Using various measuring aids, KNHANES collected the 24-hour recall at the participants’ homes [45].

FiLWHEL women without the 24-hour recall data or those who had an implausible energy intake (> 2 standard deviations above or below the log-transformed mean energy intake) were excluded, resulting in a total of 474 women. We categorized age into 20-<35, 35-<40, 40-<45, and 45-57 years and randomly selected KNHANES participants (2012-2014) from the same age category of Filipino married women (1:1 ratio) who had 24-hour recall data and whose energy intake was within the reasonable range (within 2 standard deviations of the log-transformed mean energy intake). We used a random seed in SAS and randomly selected one Korean married woman with the same age category per Filipino married woman. For example, for a Filipino married woman in the age range of 35-<40 years old, we also randomly selected a Korean married woman that was in the same age range of 35-<40 years old. The final number of participants included those who were pregnant (*n*=17 in FiLWHEL and 21 in KNHANES) or lactating (*n*=52 in FiLWHEL and 41 in KNHANES) at the time of data collection.

### Dietary diversity and food variety score

We used the DDS and FVS. DDS is defined as the number of food groups consumed over a 24-hour period [28, 30, 50]. FVS pertains to the reported number of different food items eaten over a given period [50, 51]. For the DDS, the 24-hour recall database of FiLWHEL and KNHANES allowed us to assign ingredients or food items to their appropriate food groups for foods that had different mixtures. We developed twelve food groups based on an agricultural commodity [28], similarities in nutrient composition, or uses in the diet [30]. The twelve food groups are as follows: (1) grains and tubers, (2) red meat, (3) poultry, (4) fish, (5) other seafood, (6) legumes, seeds, and nuts, (7) eggs, (8) dairy, (9) leafy vegetables, (10) other vegetables, (11) fruits, and (12) others. The grain and tuber group included all grains and tuber products. Red meat, poultry, fish, and other seafood included all processed products. The dairy group included all milk and milk products. The fruit and vegetable groups included all fresh, cooked, canned, frozen, dried, and juice products. Other vegetables included all other vegetables except for the leafy vegetables, which has its own group in our study. For the purpose of this study, we included only the basic food commodities, except for a few foods in which the ingredients were not disaggregated. The food group ‘others’, which includes condiments, sauce, coffee, tea, fats and oils, desserts, and others, were not included in the DDS calculations since most of them were either consumed in very small quantities or composed mainly of fat and simple carbohydrates or high sodium in the case of snacks [52]. We calculated the total DDS for each participant using the set of eleven food groups. Each food group received a diversity score of ‘1’. We used an all-inclusive DDS (i.e., DDS all) that was calculated without a minimum food group intake (independent of the quantity consumed) and DDS type that applies a 10 g minimum (i.e., DDS 10 g) to any of the food groups. We used two different minimum quantities to examine whether the results were consistent and robust. It has been suggested that using a 10 g minimum can improve the prediction of low nutrient adequacy [53], and the association of DDS with nutritional or disease status could vary by how the minimum quantity of DDS was determined. On the other hand, FVS simply counts all the food items. We used the dish-based approach in which we did not count the food items by ingredient but rather by the whole food [29]. For example, if the participant ate spaghetti, then we counted it as one point without considering the different ingredients that belong to the different food groups. In this scoring type, all food items were included, and those that were consumed multiple times during the period were counted only once. For this analysis, the range of food items consumed over the 24-hour period in FiLWHEL and KNHANES were 1-17 and 2-34, respectively.

### Probability of Adequacy

We determined the probability of adequacy (PA) of nutrient intake by using the probability approach [54, 55]. The National Research Council developed this approach for assessing the prevalence of nutrient inadequacy among groups [56]. We calculated the PA for each participant among nine nutrients (i.e., vitamins A, B-1, B-2, C, calcium, iron, protein, niacin, and phosphorus) using the “probnorm” function in SAS [57]. In this study, nutrient intakes were derived from foods only. The equation for each nutrient in every individual is as follows: PA=PROBNORM [(estimated intake-EAR)/SD]; and SD=RNI-EAR/2 [28, 58–60]. We used the estimated average requirement (EAR) and reference nutrient intake (RNI) values from the Dietary Reference Intakes for Koreans 2015 [61]. The mean probability of adequacy (MPA) of nutrient intake for each participant is the average of the PA for the nine nutrients. We did not adjust the effect of day-to-day variation upon calculation of the PA since we had only a single-day information. We based the selection of the nine nutrients on these two rationales: if the nutrient has an EAR value and if a reasonable nutrient database for the Korean diet exists.

### Statistical Analysis

For comparison, we calculated the prevalence and means. We used McNemar’s test for categorical variables. For continuous variables, data were analyzed by Student's paired t-test, if normality was met after a variable was log-transformed, or Wilcoxon Signed Rank test. When calculating the probability of nutrient adequacy, we applied the specific EAR for pregnant or lactating women. We used a significance level of 0.05 for all analyses. We performed all analyses using SAS version 9.4 (SAS Institute, Inc., Cary, NC, USA).

## Results

In comparison to Korean women, Filipino women were more likely to have a higher body mass index (23.6 ± 3.8 kg/m^2^ vs. 22.8 ± 3.7 kg/m^2^) and have a greater than high school education (67.3% vs. 51.9%). On the other hand, the Filipino women group had a lower number of individuals who had engagement in vigorous exercise (17.9% vs. 18.4%), ever drank alcohol (70.3% vs. 85.4%) and ever smoked (8.7% vs. 11.4%) than the Korean women group (Table 1).

**Table 1:** Baseline characteristics between women in the FiLWHEL and KNHANES (*n*=474)^a^

	FiLWHEL	KNHANES	*P-*value^b^
Total individuals	474	474	
Age			Matched
20-34	252 (53.2)	252 (53.2)	
35-39	101 (21.3)	101 (21.3)	
40-44	62 (13.1)	62 (13.1)	
45-57	59 (12.4)	59 (12.4)	
BMI^c^ (kg/m^2^)	23.6 ± 3.8^d^	22.8 ± 3.7	0.001
Education			<0.001
High school or less	153 (32.3)	181 (38.2)	
Associate/College or more	319 (67.3)	246 (51.9)	
Missing/Do not know	2 (0.4)	47 (9.9)	
Vigorous exercise			<0.001
No	379 (80.0)	342 (72.1)	
Yes	85 (17.9)	87 (18.4)	
Missing/Do not know	10 (2.1)	45 (9.5)	
Alcohol intake			<0.001
Never	137 (28.9)	28 (5.9)	
Past and current	333 (70.3)	405 (85.4)	
Missing/Do not know	4 (0.8)	41 (8.7)	
Smoking status			<0.001
Never	429 (90.5)	379 (80.0)	
Past and current	41 (8.7)	54 (11.4)	
Missing/Do not know	4 (0.8)	41 (8.6)	

*Abbreviations*: *FiLWHEL* Filipino Women’s Diet and Health Study, *KNHANES* Korea National Health and Nutrition Examination Survey, *BMI* body mass index

^a^Values are n (%) unless otherwise specified. Age category-matching was taken into account

^b^Based on McNemar’s tests for categorical variables and Student’s paired t-tests for log-transformed BMI

^c^Missing data on BMI for 3 FiLWHEL and 21 KNHANES participants

^d^Mean ± SD (all such values)

**Fig. 1:**
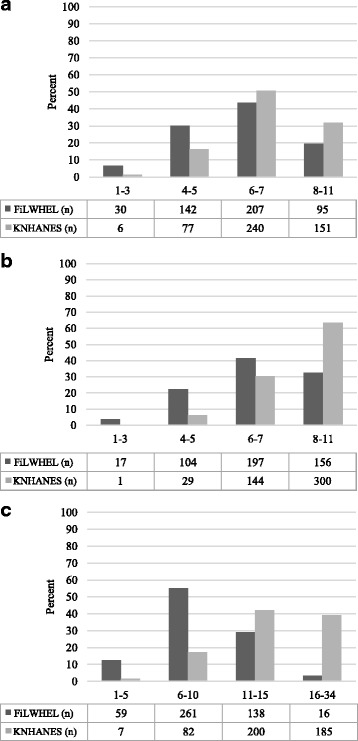
Dietary diversity score between women in FiLWHEL and KNHANES. Legend: DDS 10 g: dietary diversity score with a minimum of 10 g in each food group (**a**); DDS all: dietary diversity score with no minimum set in each food group (**b**); and FVS: count of all different food items consumed (**c**); FiLWHEL: Filipino Women’s Diet and Health Study; KNHANES: Korea National Health and Nutrition Examination Survey. Mean (±SD) of DDS 10 g, DDS all, and FVS for FILWHEL versus (v) KNHANES were 6.0 (±1.6) v 6.8 (±1.5), 6.7 (±1.7) v 7.9 (±1.4), and 9.2 (±3.3) v 14.7 (±4.9), respectively (Student’s paired t-tests *p* < 0.001, respectively). Using the McNemar’s tests, the *P*-value for the proportions of all scoring categories of DDS 10 g, DDS all, and FVS between FiLWHEL and KNHANES was <0.001

We compared the DDS and FVS between Filipino women in FiLWHEL and Korean women in KNHANES (Fig. 1). Filipino women had significantly lower mean variety scores than Korean women: DDS 10 g (6.0 ± 1.6 vs. 6.8 ± 1.5) (*p* < 0.001); DDS all (6.7 ± 1.7 vs. 7.9 ± 1.4) (*p* < 0.001); and FVS (9.2 ± 3.3 vs. 14.7 ± 4.9) (*p* < 0.001). In the DDS 10 g (Fig. 1a), both Filipino and Korean women had a high proportion in the DDS category of 6-7 (43.7% and 50.6%). In the DDS all and FVS (Fig. 1b-c), a higher concentration of Filipino women remained at the DDS category of 6-7 (41.6%) and FVS category of 6-10 (55.1%). However, Korean women were more clustered in the DDS category of 8-11 (63.3%) and FVS category of 11-15 (42.2%).

Using the DDS 10 g, in comparison with Korean women, Filipino women had the lower proportion of the following food groups (FiLWHEL vs. KNHANES) fish (42.8% vs. 50.2%), other seafood (17.7% vs. 23.2%), legumes/seeds/nuts (36.3% vs. 49.4% ), eggs (44.9% vs. 52.7%), leafy vegetables (72.2% vs. 93.0%), other vegetables (88.6% vs. 99.2%), and fruits (56.8% vs. 69.6%). This was also the same for DDS all, as follows: fish (53.6% vs. 77.4%), other seafood (25.1% vs. 43.7%), legumes/seeds/nuts (53.8% vs. 88.2%), eggs (52.1% vs. 66.2%), leafy vegetables (77.0% vs. 95.4%), other vegetables (93.9% vs. 99.4%), and fruits (60.3% vs. 74.3%) (Table 2). More than 50% of the Filipino women did not consume poultry, fish, other seafood, legumes/seeds/nuts, eggs, and dairy.

**Table 2:** Number of women (%) who consumed the specific food groups according to dietary diversity score between women in FiLWHEL and KNHANES^a^

Food groups	DDS 10 g			DDS all		
	FiLWHEL	KNHANES	*P*-value^b^	FiLWHEL	KNHANES	*P*-value^b^
Grains and tubers	472 (99.6)	473 (99.8)	0.56	474 (100.0)	473 (99.8)	-
Red meat	342 (72.2)	346 (73.0)	0.77	353 (74.5)	373 (78.7)	0.13
Poultry	153 (32.3)	114 (24.1)	0.004	162 (34.18)	118 (24.9)	0.002
Fish	203 (42.8)	238 (50.2)	0.03	254 (53.6)	367 (77.4)	<0.001
Other seafood	84 (17.7)	110 (23.2)	0.04	119 (25.1)	207 (43.7)	<0.001
Legumes/seeds/nuts	172 (36.3)	234 (49.4)	<0.001	255 (53.8)	418 (88.2)	<0.001
Eggs	213 (44.9)	250 (52.7)	0.02	247 (52.1)	314 (66.2)	<0.001
Dairy	188 (39.7)	194 (40.9)	0.69	202 (42.6)	214 (45.2)	0.43
Leafy vegetables	342 (72.2)	441 (93.0)	<0.001	365 (77.0)	452 (95.4)	<0.001
Other vegetables	420 (88.6)	470 (99.2)	<0.001	445 (93.9)	471 (99.4)	<0.001
Fruits	269 (56.8)	330 (69.6)	<0.001	286 (60.3)	352 (74.3)	<0.001

*Abbreviations*: *FiLWHEL* Filipino Women’s Diet and Health Study, *KNHANES* Korea National Health and Nutrition Examination Survey

^a^Values are n (%) of those who consumed in each food group per day. Age category-matching was taken into account

^b^Based on McNemar’s test

^-^Not applicable because FiLWHEL has a zero cell count

There were no significant differences between the mean intakes of the Filipino women and Korean women in energy, protein, and niacin (Table 3). However, there were significant differences in the mean intakes of fat, carbohydrates, calcium, iron, phosphorus, and vitamins A, B1, B2, and C. Filipino women had lower intakes of carbohydrate but higher fat intakes than Korean women. Nevertheless, for most nutrients, Filipino women had a significantly lower nutrient intake in comparison to Korean women. Filipino women had a significantly lower PAs of vitamins A, B1, and B2, calcium, iron, and phosphorous than Korean women. The PA in Filipino women was <0.50 for vitamins A, B2, and C, and calcium and >0.50 for protein, vitamin B1, iron, niacin, and phosphorus. For Korean women, only vitamin C and calcium had a PA of <0.50. The MPA across the nine nutrients was 0.55 for Filipino women and 0.66 for Korean women (*p* for difference <0.001). We found a higher proportion of Filipino women who consumed below the EARs of vitamins A, B1, and B2, calcium, iron, and phosphorus in comparison to Korean women.

**Table 3:** Intakes for selected nutrients, probability of adequate nutrient intake, and estimated average requirement between women in FiLWHEL and KNHANES^a^

Nutrients	Intake			PA^b^			Below the EAR^c^		
	FiLWHEL	KNHANES	*P*-value^d^	FiLWHEL	KNHANES	*P*-value^d^	FiLWHEL	KNHANES	*P*-value^e^
Energy (kcal)	1813.3 ± 638.6	1853.9 ± 648.8	0.32	-	-	-	307 (64.8)^f^	291 (61.4)	0.28
Fat (g)	54.5 ± 29.2	45.7 ± 27.0	<0.001	-	-	-	-	-	-
Carbohydrates (g)	257.1 ± 102.1	283.6 ± 103.9	<0.001	-	-	-	-	-	-
Protein (g)	72.6 ± 32.2	70.6 ± 35.8	0.14	0.81 ± 0.34	0.80 ± 0.34	0.22	89 (18.8)	92 (19.4)	0.80
Vitamin C (mg)	88.6 ± 85.8	105.2 ± 102.4	0.01	0.42 ± 0.46	0.48 ± 0.46	0.006	275 (58.0)	247 (52.1)	0.07
Vitamin A (ug RE)	581.2 ± 630.6	881.1 ± 925.8	<0.001	0.43 ± 0.43	0.65 ± 0.41	<0.001	276 (58.2)	166 (35.0)	<0.001
Calcium (mg)	396.2 ± 280.4	484.6 ± 333.4	<0.001	0.26 ± 0.38	0.36 ± 0.40	<0.001	352 (74.3)	313 (66.0)	0.005
Vitamin B-1 (mg)	1.31 ± 1.19	1.67 ± 0.84	<0.001	0.61 ± 0.45	0.80 ± 0.36	<0.001	186 (39.2)	92 (19.4)	<0.001
Vitamin B-2 (mg)	1.05 ± 0.60	1.32 ± 0.67	<0.001	0.42 ± 0.45	0.62 ± 0.44	<0.001	276 (58.2)	178 (37.6)	<0.001
Iron (mg)	12.7 ± 8.4	15.5 ± 8.6	<0.001	0.51 ± 0.44	0.67 ± 0.41	<0.001	234 (49.4)	158 (33.3)	<0.001
Niacin (mg)	16.9 ± 9.2	16.5 ± 8.4	0.43	0.70 ± 0.41	0.70 ± 0.39	0.74	144 (30.4)	139 (29.3)	0.72
Phosphorus (mg)	949.5 ± 440.4	1057.9 ± 448.9	<0.001	0.80 ± 0.36	0.89 ± 0.27	<0.001	91 (19.2)	51 (10.8)	<0.001
MPA	-	-	-	0.55 ± 0.28	0.66 ± 0.26	<0.001	-	-	-

*Abbreviations*: *FiLWHEL* Filipino Women’s Diet and Health Study, *KNHANES* Korea National Health and Nutrition Examination Survey, *PA* probability of adequate nutrient intake, *EAR* estimated average requirement, *EER* estimated energy requirement, *MPA* mean probability of adequate nutrient intake, *RNI* recommended nutrient intake

^a^Values are mean ± SD unless otherwise indicated. Age category-matching was taken into account

^b^The PA for each nutrient’s z-score, estimated intake-EAR/SD, was determined by using the “probnorm” statistical function in SAS [57, 58]

^c^EER for energy and EAR for the rest of nutrients

^d^Based on Wilcoxon Signed Rank Test

^e^Based on McNemar’s test

^f^Values are n (%)

^-^Not applicable

We found that Filipino women who received a higher education tended to have a higher DDS compared to those who had high school or less. Women who were past and current alcohol drinkers had a tendency of having a lower DDS compared to never drinkers (Additional file 1: Table S1).

## Discussion

The Filipino women in our study were less likely to consume a variety of food groups than Korean women. In other words, Filipino women married to Korean men had a lower DDS in comparison to Korean women. In particular, we observed a lower DDS of fish, other seafood, legumes/seeds/nuts, eggs, vegetables, and fruits in Filipino women compared to Korean women.

Several previous studies across countries have reported on dietary diversity. A cross-sectional study of Tehranian women reported a mean DDS of 6.0, an MPA of 50.1%, and a statistically significant correlation of 0.6 between MPA and DDS [60]. In the 1994-1996 Continuing Survey of Food Intakes by Individuals (CSFII 94-96) of a US population, the mean of total variety (conceptually similar to the mean DDS) was 7.3, and the MPA was 57.7%. That study reported that the correlation between MPA and total variety was 0.68 [28]. We used similar methods as these studies that included the use of a minimum in counting dietary diversity. They used at least a one-half serving, and one of our variety scoring types used 10 g as the minimum. Foote et al. (CSFII 94-96) also used a disaggregated database such that a food item or an ingredient could be assigned easily into the food group to which it belongs. For FVS, we used the dish-based style, which simply counts all food items. A cross-sectional study in Burkina Faso, West Africa examined the FVS among mothers (*n*=691) and reported the mean FVS of 8.3 food items [62], which was slightly lower than our results (mean FVS = 9.2 food items).

A report from the 7^th^ National Nutrition Survey in the Philippines showed that Filipino adults (20-59 years) consumed an average of 91 g per day of vegetables [20]. In our analysis, we found that the total vegetable intake of Filipino and Korean women was 208.4 g per day and 332.8 g per day (*p* <0.001) (data not shown), respectively. If we compare our data to those of Filipino adults in the Philippines, the vegetable intake of Filipino women in Korea was more than twice as high. The mean total meat intake for Filipinos in the Philippines was 95 g per day in the 7^th^ National Nutrition Survey [20]. We found that Filipino women in our study consumed 143.4 g of total meat, which was a 51% higher intake compared to Filipinos from the National Nutrition Survey in the Philippines. However, our results focused only on married women, whereas the population of the Filipino National Nutrition Survey included both adult men and women. Nevertheless, our sample’s age range was quite comparable (FiLWHEL: 20-57 years and the Filipino National Nutrition Survey: 20-59 years).

In our study, except for protein, vitamin C, and niacin which were similar among the two groups, the rest of the nutrients’ PA values were significantly higher in Korean than Filipino women. Among the selected nine nutrients in the Filipino women group in our study, only protein, niacin, and phosphorus had a mean PA of ≥ 0.70, and calcium, iron, and vitamins A, B1, B2, and C had a PA of ≤0.61. The lowest PA was for calcium (0.26 for Filipino women and 0.36 for Korean women), which was the same as the pattern for Korean women compared with the other selected nutrients. The National Nutrition Survey in the Philippines reported a relatively high proportion of Filipino adults who could meet the EARs of niacin (89%) and protein (64%) but a low proportion (<40%) of those who could meet the EARs of calcium, iron, and vitamins A, B1, B2, and C [20]. Similar to our analysis, the report of the Filipino National Nutrition Survey indicated that calcium also had the lowest adequacy (9.6%). If we compared Filipino adults in the Philippines using the report from the 7^th^ National Nutrition Survey and the nutrient adequacy of the nutrients that we examined (except for niacin and protein), Filipino women in Korea were intermediate between Filipino adults in the Philippines and Korean women. This finding may suggest that there could be changes in the dietary pattern among the Filipino women after they moved to Korea.

Kant et al. found in a cross-sectional analysis of a subsample from the Third National Health and Nutrition Examination Survey, 1988-1994 (*n*=8719) that the DDS for the recommended food groups and the other two dietary pattern indexes (Healthy Eating Index and Recommended Foods Score) were independent positive predictors of the serum concentration of nutrients. These include vitamins C, E, folate, and all carotenoids, with the exception of lycopene, which were inversely associated with body mass index, and biomarkers of disease risks, including serum homocysteine, C-reactive protein, plasma glucose, and hemoglobin A1C [63]. Cross-sectional analyses in the Tehran Lipid and Glucose Study among adults over 18 years found that those who had a higher DDS had lower odds of having cardiovascular risk factors [64, 65]. These results also hold in the same manner for female Iranian youths aged 18-28. DDS was inversely associated with obesity [66]. Several case-control studies have shown that total diversity was inversely associated with gastric, oral and pharyngeal, squamous cell esophageal, colorectal, and bladder cancers [33–37]. Furthermore, in a prospective study, total dietary diversity was associated with a 30% lower risk of developing type 2 diabetes [32]. Based on these previous findings, it is therefore suggestive that DDS and FVS, though simple as they appear, are useful in assessing dietary diversity and further diet-disease analysis.

We observed that high education levels were positively associated with DDS in the FiLWHEL study, suggesting that a better socioeconomic status may help Filipino immigrant women in pursuing a healthy diet. The importance of nutrition education focusing on eating a variety of foods should be emphasized at the individual and community levels for Filipino immigrant women in Korea.

This is, to our knowledge, the first study comparing nutrient intake between Filipino immigrant women in Korea and Korean women using three types of diversity scoring (DDS all, DDS 10 g, and FVS). This study has several limitations. First, we collected the 24-hour recall data for a single day, which does not represent the usual daily intake due to day-to-day variations [67]. Second, FiLWHEL is composed of Filipino women who were residents in some selected regions in Korea, which may limit the generalizability of our findings to all Filipino women in Korea. Third, the matching between the Filipino and Korean women samples was based on an age criterion only. Age is an important covariate associated with nutrient adequacy; however, using one matching variable, we cannot exclude the possibility of selection bias between the two samples.

## Conclusions

We found that married Filipino women in Korea had lower levels of dietary diversity in comparison to married Korean women. These diversity levels were reflected in a low nutrient adequacy. Filipino women were less favorable to fish, other seafood, legumes/seeds/nuts, eggs, vegetables, and fruits in comparison to Korean women. Therefore, we recommend a culturally appropriate nutrition education to be carried out emphasizing the importance of eating a variety of foods among married Filipino women in Korea.

## Additional file

Additional file 1: **Table S1.** Baseline characteristics according to dietary diversity and food variety scores in the FiLWHEL. (DOCX 20 kb)

## Abbreviations

CVD: Cardiovascular disease
DDS 10 g: with a minimum of 10 g in each food group
DDS all: no minimum set in each food group
DDS: Dietary diversity score
EAR: Estimated average requirement
FVS: Food variety score
FiLWHEL: Filipino Women’s Diet and Health Study
KNHANES: Korea National Health and Nutrition Examination Survey
MPA: Mean probability of adequate nutrient intake
PA: Probability of adequate nutrient intake
