# Supplementary material for: Dietary diversity and nutritional adequacy among married Filipino immigrant women: The Filipino Women’s Diet and Health Study (FiLWHEL)
Source: BMC Public Health. 2018 Mar 15;18:359. doi: 10.1186/s12889-018-5233-z (PMC5856319; doi:10.1186/s12889-018-5233-z)
Supplement: Supplementary file 1 — Table S1. Baseline characteristics according to dietary diversity and food variety scores in the FiLWHEL. (DOCX 20 kb) [file 12889_2018_5233_MOESM1_ESM.docx]

**Supplemental table 1.** Baseline characteristics according to dietary diversity and food variety scores in the FiLWHEL^a^

|  | DDS 10 g | | | |  | DDS All | | | |
| --- | --- | --- | --- | --- | --- | --- | --- | --- | --- |
|  | 1-3 | 4-5 | 6-7 | 8-11 |  | 1-3 | 4-5 | 6-7 | 8-11 |
| Total individuals | 31 | 142 | 206 | 92 |  | 17 | 106 | 195 | 153 |
| Age, years |  |  |  |  |  |  |  |  |  |
| 20-34 | 15 (48.4) | 78 (54.5) | 113 (54.6) | 46 (49.5) |  | 7 (41.2) | 57 (53.8) | 115 (58.4) | 73 (47.4) |
| 35-39 | 8 (25.8) | 27 (18.9) | 39 (18.8) | 27 (29.0) |  | 4 (23.5) | 19 (17.9) | 35 (17.8) | 43 (27.9) |
| 40-44 | 6 (19.4) | 18 (12.6) | 32 (15.5) | 6 (6.5) |  | 6 (35.3) | 16 (15.1) | 23 (11.7) | 17 (11.0) |
| 45-57 | 2 (6.4) | 20 (14.0) | 23 (11.1) | 14 (15.0) |  | 0 (0.0) | 14 (13.2) | 24 (12.2) | 21 (13.6) |
| BMI, kg/m^2^ | 24.1±3.3^b^ | 23.6±3.8 | 23.7±4.0 | 23.1±3.3 |  | 23.7±3.6 | 23.5±3.5 | 23.5±3.9 | 23.7±9.4 |
| Education |  |  |  |  |  |  |  |  |  |
| High school or less | 14 (45.2) | 51 (35.7) | 61 (29.6) | 27 (29.3) |  | 5 (29.4) | 46 (43.4) | 65 (33.2) | 37 (24.2) |
| Associate/College or more | 17 (54.8) | 92 (64.3) | 145 (70.4) | 65 (70.7) |  | 12 (70.6) | 60 (56.6) | 131 (66.8) | 116 (75.8) |
| Alcohol intake |  |  |  |  |  |  |  |  |  |
| Never | 8 (25.8) | 30 (21.0) | 64 (31.2) | 35 (38.5) |  | 4 (23.5) | 26 (24.5) | 52 (26.7) | 55 (36.2) |
| Past and current drinkers | 23 (74.2) | 113 (79.0) | 141 (68.8) | 56 (61.5) |  | 13 (76.5) | 80 (75.5) | 143 ()73.3 | 97 (63.8) |
| Vigorous exercise |  |  |  |  |  |  |  |  |  |
| No | 26 (86.7) | 119 (83.8) | 157 (77.0) | 77 (87.5) |  | 14 (87.5) | 88 (83.8) | 153 (78.9) | 124 (83.2) |
| Yes | 4 (13.3) | 23 (16.2) | 47 (23.0) | 11 (12.5) |  | 2 (12.5) | 17 (16.2) | 41 (21.1) | 25 (16.8) |
| Smoking status |  |  |  |  |  |  |  |  |  |
| Never | 26 (83.9) | 133 (93.0) | 187 (91.2) | 83 (91.2) |  | 14 (82.4) | 97 (91.5) | 180 (92.3) | 138 (90.8) |
| Past and current drinkers | 5 (16.1) | 10 (7.0) | 18 (8.8) | 8 (8.8) |  | 3 (17.6) | 9 (8.5) | 15 (7.7) | 14 (9.2) |

^a^ Values are n (%) unless otherwise specified. Except for age, numbers may not add to the total N (474) due to missing information. ^b^ Mean ± SD (all such values).
